# Age-related dissociation of N400 effect and lexical priming

**DOI:** 10.1038/s41598-020-77116-9

**Published:** 2020-11-20

**Authors:** Hannes O. Tiedt, Felicitas Ehlen, Fabian Klostermann

**Affiliations:** 1 Department of Neurology, Motor and Cognition Group. Campus Benjamin Franklin (CBF), Charité – Universitätsmedizin Berlin, Hindenburgdamm 30, 12203 Berlin, Germany; 2 Present Address: Department of Psychiatry, Jüdisches Krankenhaus Berlin, Heinz-Galinski-Straße 1, 13347 Berlin, Germany; 3 Berlin School of Mind and Brain, Humboldt-Universität zu Berlin, Unter den Linden 6, 10099 Berlin, Germany

**Keywords:** Cognitive ageing, Neuroscience, Cognitive neuroscience, Language

## Abstract

The use of contextual information is an important capability to facilitate language comprehension. This can be shown by studying behavioral and neurophysiological measures of accelerated word recognition when semantically or phonemically related information is provided in advance, resulting in accompanying attenuation of the respective event-related potential, i.e. the N400 effect. Against the background of age-dependent changes in a broad variety of lexical capacities, we aimed to study whether word priming is accomplished differently in elderly compared to young persons. 19 young (29.9 ± 5.6 years) and 15 older (69.0 ± 7.2 years) healthy adults participated in a primed lexical decision task that required the classification of target stimuli (words or pseudo-words) following related or unrelated prime words. We assessed reaction time, task accuracy and N400 responses. Acceleration of word recognition by semantic and phonemic priming was significant in both groups, but resulted in overall larger priming effects in the older participants. Compared with young adults, the older participants were slower and less accurate in responding to unrelated word-pairs. The expected N400 effect was smaller in older than young adults, particularly during phonemic word and pseudo-word priming, with a rather similar N400 amplitude reduction by semantic relatedness. The observed pattern of results is consistent with preserved or even enhanced lexical context sensitivity in older compared to young adults. This, however, appears to involve compensatory cognitive strategies with higher lexical processing costs during phonological processing in particular, suggested by a reduced N400 effect in the elderly.

## Introduction

Language capacities are age-dependent with approximately two decades of acquisition^1–4^ and a gradual decline in advanced age^5,6^. However, the latter seems true for language production rather than comprehension. For example, older adults perform worse in naming and verbal fluency tasks than younger persons and typically report difficulties in word finding with a frequent occurrence of ‘tip-of-the-tongue’ states^7–10^, but their semantic and verbal knowledge is preserved or even increased^11–13^. On the other hand, language comprehension in elderly persons might suffer from non-cognitive, sensory changes, such as progressive hearing problems^14^. Lower hearing acuity may impede the differentiation of speech from noise (‘cocktail-party’ situation) and single words within the acoustic stream of lexical information^15,16^. In particular, older adults exhibit greater difficulties with word recognition and the differentiation of words with a greater number of phonetically similar words (i.e. with higher ‘neighborhood density’) but show stronger benefits from semantic context than younger participants at the same time^17,18^. Likewise, the negative effect of mild to moderate hearing loss on word discriminability can be diminished by semantic context^19,20^. These findings are consistent with the hypothesis that elderly persons might rely on contextual information to disambiguate speech signals more strongly than younger persons to compensate for sensory changes, whereas lexical and semantic processing appear to be relatively intact^21^. This view of ‘semantic sparing’ has also been supported by observations of preserved or even larger semantic priming effects (‘hyperpriming’) in older adults^22,23^. In keeping with a prominent role of contextual information for word recognition in older age, several investigations using event-related potentials (ERPs) have also suggested age-related changes of lexico-semantic processes underlying the N400 component, e.g.,^24–27^.

The N400 component is a negative polarity shift at central-posterior electrode-sites occurring between 200 and 600 ms following the presentation of a broad range of stimulus-types, which has been studied most intensively as an index of language processing; for reviews of the N400 see e.g.^28–30^. The amplitude of the N400 is sensitive to manipulations of lexical characteristics of words such as their frequency of occurrence^31,32^ and neighborhood density^33^. Most consistently, however, the N400 has been found to be reduced upon words that are embedded in a congruent compared to an implausible language context such as sentence fragments^34,35^ or a discourse consisting of several sentences^36,37^. This amplitude reduction (i.e., the ‘N400-effect’) is observed in a similar way to target words that are semantically related to preceding single words^38,39^, corresponding to the behavioral result of context-facilitated target recognition in word priming^40^. A common interpretation of the N400 in this regard is that it reflects the integration of linguistic context with incoming sensory information during lexical processing to gain access to memory representations^28,41^. However, N400 amplitude reductions have also been observed to phonologically or orthographically related word pairs^42–45^ as well as during pseudo-word or non-word processing^46,47^. Therefore, several authors have focused their interpretation of the N400 effect more towards predictive—including semantic—processing and lexical retrieval, e.g.^29,30,48,49^. During language comprehension, prediction based on context can reduce the amount of sensory information required for the successful recognition of words^50^ and is thought to occur at multiple levels during the processing of speech signals^51^.

Regarding cognitive ageing, studies in older adults have consistently shown significantly smaller or even absent N400 amplitude reductions upon semantic congruency^25,52,53^, contextual constraint^24,54–56^, or lexically associated word-pairs within sentences^57^. Although less consistent (see for example^55^), the peak-latency of the N400 is typically delayed in older compared with younger adults. Of note, N400 components elicited by single words without lexical context have been demonstrated to show no significant^58^ or only little^59^ modulation by increasing age; the latter study reported decreased N400 amplitudes to real words in older adults with no age-difference regarding pseudo-words. Ageing thus appears to be more specifically associated with a gradual decrease of the N400 effect, i.e. the amplitude reduction elicited by semantic congruity or constraining context^25^. These observations have been commonly interpreted as an indication of an age-dependent decline of the ability to facilitate semantic access during language comprehension through prediction based on linguistic context; for reviews see^30,60,61^. This view, however, is at odds with the aforementioned behavioral data suggesting preserved or even larger context sensitivity in older adults based on word priming experiments.

We therefore sought to analyze the age-dependency of lexical priming together with potentially accompanying N400 modulations in a primed auditory lexical decision task (LDT), providing easily quantifiable behavioral parameters referable to the neurophysiological data. In doing so, we first of all expected to confirm the seemingly contradictory age-dependent dissociation of preserved (or larger) priming and reduced N400 effect in one task. This would be of interest in regard to (i) the processing of age-dependent context sensitivity and (ii) an adjusted concept of the N400 component. At the same time, any other result pattern out of the possible constellations (absent / reduced / preserved / enhanced priming—enhanced / preserved / reduced / absent N400 effect) would be informative of these issues.

During ERP acquisition, participants listened to spoken item-pairs consisting of a prime word and a target-stimulus (real word or pseudo-word). Each prime word was followed by either (i) a semantically related word (e.g., TIGER—LION), (ii) a phonemically related word (i.e., rhymes such as RAMP—LAMP), (iii) an unrelated word (e.g., TABLE—WIND), (iv) a phonemically related pseudo-word (e.g., LIGHT—VIGHT), or (v) an unrelated pseudo-word (e.g., APPLE—LORK). We planned to analyze LDT performance (reaction times and priming effects) and accuracy (error-rates) in addition to N400 latency and amplitudes (measured as the mean voltage 350–700 ms post-stimulus). Further details about the experimental procedures are provided in the "Methods" section at the end of the article.

## Results

### Behavioral results

For group mean values of RTs, priming effects, and error-rates, see Table 1.

**Table 1:** Behavioral results.

	Old	Young
**Lexical decision time (ms)**		
Unrelated	1076 (± 74)	1018 (± 71)
Related	926 (± 88)	923 (± 71)
**Priming effect (ms)**		
Unrelated–related	150 (± 43)	95 (± 40)
Unrelated–sem. rel	120 (± 86)	81 (± 61)
Unrelated–phon. rel	179 (± 58)	109 (± 51)
**Error rate (%)**		
Total	6.7 (± 6.4)	3.5 (± 4.5)
Omission	5.6 (± 5.4)	2.0 (± 2.1)
Comission	7.7 (± 8.8)	3.27 (± 3)
Unrelated	8.4 (± 6.9)	1.7 (± 3.0)
Sem. related	0.9 (± 3.4)	2.1 (± 3.9)
Phon. related	7.6 (± 10.3)	2.5 (± 4.6)

Lexical decision times are RTs upon target words measured from stimulus onset until button press; RTs to related target words were collapsed across the semantic and phonemic subtasks. Priming effects were calculated by subtraction of RTs upon (semantically and phonemically) related and unrelated target words. Omission errors were defined as missed button press upon real target words; commission errors as wrong button press upon pseudo-words. Values are Means (± S.D.). For comparisons between groups and task categories see "Results".

#### Lexical decision task performance

RTs to unrelated and related (i.e., averaged across semantic and phonemic relatedness) words were entered into a mixed repeated measures ANOVA with *relatedness* (related / unrelated) and *group* (old / young). This yielded a significant main effect of *relatedness* (F(1,32) = 292.822; *p* < 0.001; η_p_^2^ = 0.901), confirming faster recognition of target words related to the preceding primes. Furthermore, there was a significant interaction between *relatedness* and *group *(F(1,32) = 14.797; *p* = 0.001; η_p_^2^ = 0.316) but no significant main effect of *group* (*p* = 0.425). Further post-hoc analysis of this interaction by group comparisons of RTs revealed that RTs upon related words did not differ as a function of age (*p* = 0.930), yet RTs in the unrelated condition were longer among older participants (*p* = 0.028). Note that the latter did not reach a Bonferroni-adjusted significance level (*p* < 0.025).

Furthermore, the size of semantic and phonemic priming effects was computed by subtraction of RTs upon related target words (per condition) from RTs to unrelated target words. Priming effects were compared by means of a mixed repeated measures ANOVA with *priming* (semantic / phonemic) and *group* as above. The analysis of priming effects yielded a significant main effect of *group* (F(1,32) = 14.797; *p* = 0.001, η_p_^2^ = 0.316) due to overall larger priming effects in the older group. The main effect of *priming* was also significant (F(1,32) = 6.538; *p* = 0.016; η_p_^2^ = 0.170), indicating larger phonemic than semantic priming effects in all participants as there was no significant interaction between both factors.

#### Lexical decision task accuracy

Error-rates were not normally distributed and were therefore compared between groups by means of the Wilcoxon-test. The overall error-rate was significantly lower in younger adults (*p* = 0.012). A group comparison of the distinct error-types upon words or pseudo-words regardless of relatedness (omission vs. commission errors, respectively) revealed a higher rate of omission errors upon real words among older adults (*p* = 0.009) with a smaller and non-significant difference for commission errors upon pseudo-words (*p* = 0.111; adjusted *p* < 0.025). The group-difference for omission errors mainly emerged from errors upon unrelated word-pairs (*p* = 0.001) while being smaller and not significant for related word-pairs (phonemic: *p* = 0.083; semantic: *p* = 0.372; adjusted *p* < 0.017).

### Event-related potentials (ERPs)

#### N400 amplitude

**Figure 1:**
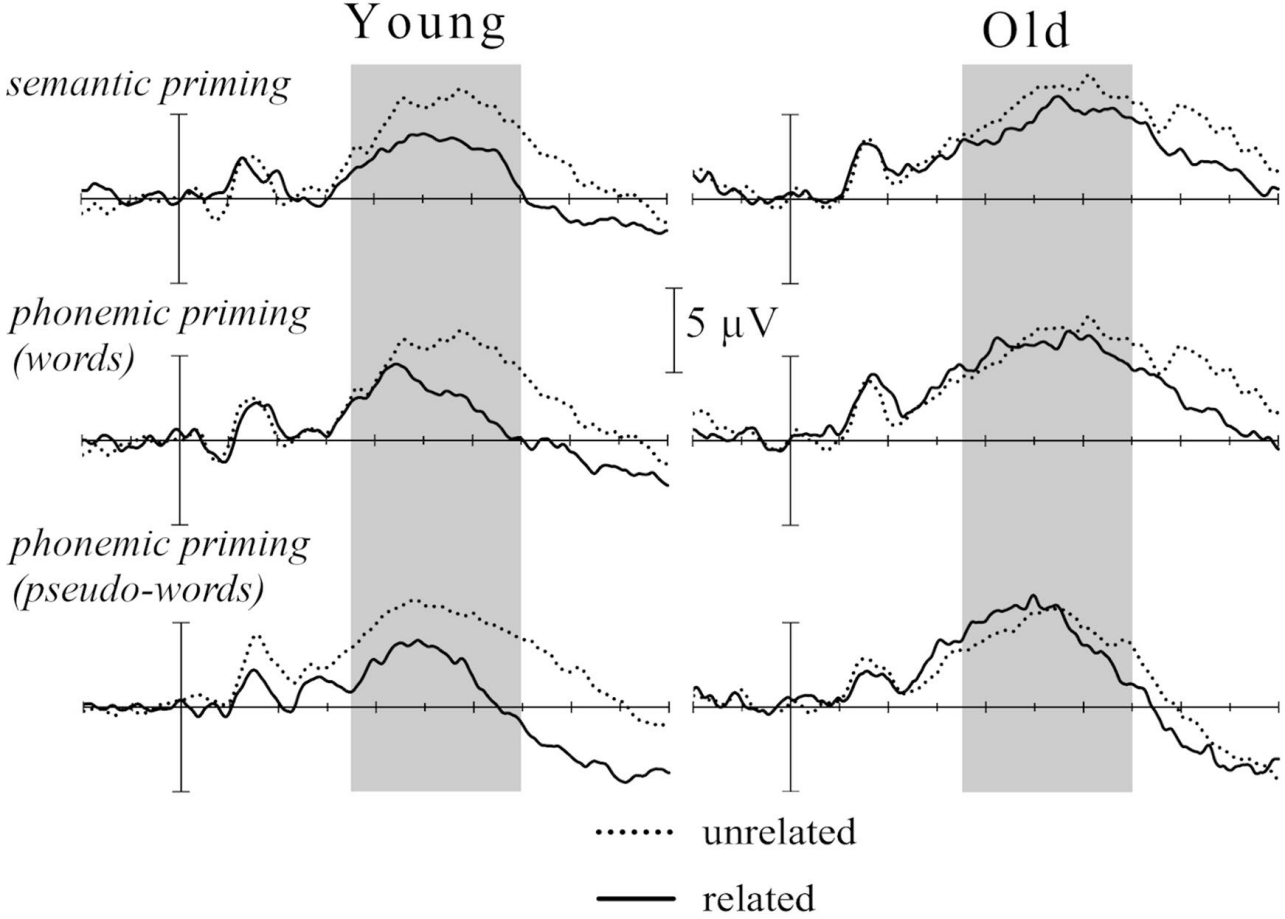
ERP upon target-stimuli. Grand-averaged ERP at electrode-site Cz between −200 and 1000 ms relative to target-stimulus onset by priming condition and for both age groups separately. The N400 time-window used for averaging ERP voltage (between 350 and 700 ms post-stimulus) is marked in grey. Negative values are plotted up.

Across all participants, target-stimuli elicited a negative ERP-component (N400) between 300–800 ms post-stimulus, preceded by an earlier N1-P2 complex, which was largest at electrode-site Cz. Grand-averaged ERPs upon all target stimuli by subcategory are depicted in Fig. 1. Group means for ERP mean voltage and fractional area latency can be taken from Table 2. It was not possible to analyze effects of electrode localization, prime-target relatedness, and group in a mixed model ANOVA for both semantic and phonemic priming since the Box’s test indicated no homogeneity of covariances (semantic priming: *p* = 0.003; phonemic priming: *p* < 0.001). Therefore, we conducted mixed model ANOVAs for the N400 amplitudes obtained at each midline electrode separately (Fz, Cz, Pz). At electrode localizations Fz and Pz, this yielded significant main effects of *relatedness* and no further significant main effects or interactions, therefore we do not report these results any further and only include the results obtained at Cz in the following.

**Table 2:** N400 mean voltage and fractional area latency.

	N400 Amplitude (µV)		N400 latency (ms)	
	Old	Young	Old	Young
**Words**				
Unrelated	−6.41 (± 3.87)	−5.22 (± 4.28)	535 (± 16)	533 (± 25)
Sem. related	−5.05 (± 3.44)	−3.17 (± 5.1)	537 (± 20)	526 (± 31)
Phon. related	−5.37 (± 4.29)	−3.02 (± 4.42)	529 (± 23)	502 (± 40)
**Pseudo-words**				
Unrelated	−5.22 (± 3.81)	−5.20 (± 3.84)	530 (± 21)	520 (± 16)
Phon. related	−5.36 (± 3.63)	−2.27 (± 4.95)	514 (± 20)	511 (± 29)

ERPs mean voltage between 350–700 ms post-stimulus (N400) and mean fractional area latencies in both age-groups obtained at electrode Cz; standard deviations are given in brackets.

For phonemic priming, the ANOVA yielded a slightly larger effect of *relatedness* (F(1,32) = 9.614, p = 0.004, η_p_^2^ = 0.231) as well as a significant interaction of *relatedness* and *group* (F(1,32) = 4.783, p = 0.036, partial η^2^ = 0.130). A post-hoc comparison between phonemically related and unrelated target-stimuli (i.e., collapsed across target words and pseudo-words) revealed that this difference was only significant (adjusted *p*-value < 0.025) among younger participants (related: −2.64 ± 3.98 µV, unrelated: −5.21 ± 3.73 µV; *p* = 0.004) and not in the older age group (related: −5.37 ± 3.37 µV, unrelated: −5.81 ± 3.30 µV; *p* = 0.370).

For semantic priming, the ANOVA yielded a significant main effect of *relatedness* (F(1,32) = 5.223, *p* = 0.029, η_p_^2^ = 0.140) without a significant main effect of *group* or an interaction between both factors.

#### N400 latency

Repeating this ANOVA for N400-latency at Cz for phonemic priming yielded a significant main effect of *relatedness* (F(1,32) = 20.011, p < 0.001, η_p_^2^ = 0.385) with shorter latency upon related than unrelated target-stimuli. The comparison between groups did not reach significance (*p* = 0.098), but there was a significant interaction between *relatedness*, *category,* and *group* (F(1,32) = 4.400, *p* = 0.044, η_p_^2^ = 0.121). This interaction is best explained by a prolonged N400 latency upon phonemically related words in older compared to younger adults (*p* = 0.029), which did not reach statistical significance after Bonferroni correction (adjusted *p*-level < 0.0125). The ANOVA for semantic priming did not yield significant main effects of *relatedness* (*p* = 0.646), *group* (*p* = 0.364), or the interaction between both factors (*p* = 0.364).

## Summary of results

To summarize, all participants showed typical behavioral priming effects with faster lexical decisions upon phonemically or semantically related than unrelated word-pairs. Older participants responded more slowly to unrelated target words and showed larger priming effects than younger adults. Furthermore, older adults had lower task accuracy with higher error-rates in total and upon unrelated word-pairs in particular. With respect to the N400 effect, younger participants showed decreased responses elicited by related items across all task subcategories, whereas this effect was considerably smaller in older adults. Here, the N400 effect elicited by phonemic priming at electrode Cz was virtually absent for pseudo-words and words alike. There was no statistical group difference with respect to semantic priming, which, however, elicited a smaller N400 effect in older adults as can be taken from Fig. 1 and Table 2. Notably, these age-effects presented at Cz being the electrode-site showing the largest N400 response and were, on the other hand, not detected at Fz and Pz.

## Discussion

Similar to the known context-related N400 effects^38^, our results show an age-dependent modulation of the N400 effect elicited by word priming. The results in older adults can be interpreted as a dissociation of linguistic context effects at the electrophysiological and behavioral level: whereas in young participants both N400 amplitude reductions and task performance revealed corresponding context sensitivity, older adults exhibited considerably smaller N400 modulation by stimulus relatedness despite even larger behavioral priming effects. When viewed in isolation, the results are consistent with previous investigations reporting reduced N400 modulations in older adults^24,25,27,52,55,56,61,62^ and behavioral studies that demonstrated preserved sensitivity to lexical context in older adults^18,22,63,64^. The pattern of behavioral and ERP results combined, however, seems difficult to reconcile with the commonly suggested interpretation of reduced N400 effects in older adults as reflecting a declining ability to facilitate semantic access by predicting upcoming lexical content based on contextual information^24,26,30,54,60,61^.

A separate analysis of RTs upon the task’s subcategories revealed that larger priming effects (semantic and to a greater extent phonemic) among older adults could be traced back to slower responses upon unrelated word-pairs, whereas lexical decision times upon related word-pairs were similar in both age-groups. This observation can be interpreted as increased challenge to classify target stimuli, when no contextual information is available. This is consistent with similar results from earlier studies which have been interpreted as demonstrating greater sensitivity to lexical context in older adults^17–19^, yet it appears to be better characterized as a greater need for lexical context with increasing age. A closer examination of reduced task-accuracy observed among older adults also supports the latter interpretation: older adults made significantly more errors during classification of unrelated target-words but identified semantically related targets as accurately as younger adults, indicating a greater benefit from contextual information but also more difficulties if no context is available. Of note, previous meta-analyses have also demonstrated larger (semantic) priming effects in older as compared to young adults^22,23^. In these studies, however, the analysis was focused on priming effects calculated as the difference between RTs to either related or unrelated word-pairs, so that it remains unclear if prolonged reactions upon unrelated stimuli in particular (as observed in our participants) was associated with larger priming. An alternative account for larger priming effects in older adults has been provided by the suggestion of distinct ‘post-access’ decision making strategies based on the degree of stimulus-relatedness^21^. In this view, the degree of semantic relatedness between target words and primes could support lexical decision making by means of backward checking on stimulus-relatedness. This process, being less efficient than retrieval through semantic spreading activation, could thus slow down responses to unrelated targets. Regarding phonemic priming, a similar strategic component of lexical decisions based on phoneme overlap was suggested in addition to non-strategic automatic processing prior to lexical access^65^. A stronger reliance on such post-access strategies based on prime-target relatedness would, however, not support lexical decision making in the case of (phonemically related) pseudo-words. Thus, if larger priming effects in older adults indeed reflected a greater need for lexical context, similar findings might also be expected for the contrast between unrelated and related pseudo-word targets. Based on the current results, this question remains open as we used a go / no-go variant of the LDT, which demanded a button-press upon real words but no behavioral response upon pseudo-words. Therefore, future studies might use an LDT procedure demanding differential behavioral responses upon target words and pseudo-words to obtain comparable behavioral parameters for both categories of target stimuli.

Furthermore, findings of larger priming effects in older adults were discussed in the context of various other experimental findings and theoretical accounts, suggesting either general^23^ or process-specific^22^ cognitive slowing. Slowing of cognitive operations has also been suggested in the context of prolonged N400 peak-latencies observed in older adults in some^24,25,60^ (but not all^55^) previous studies. We did not find overall delayed N400 components, but only prolonged latencies to phonemically primed target words in older participants (which was not statistically significant after Bonferroni correction).

Smaller or absent reductions of the N400 amplitude elicited by semantic or phonemic priming of both words and pseudo-words in older adults can be related to the view of the N400 effect reflecting primarily a degree of association between stimuli as vastly *independent* from semantics^66^. A rather basic characterization of the N400 is supported by several investigations demonstrating the relevance of non-semantic aspects of word processing for modulations of the N400 elicited by phonemic and orthographic relatedness as well as associated pseudo-words and even unpronounceable non-words^42–47,67,68^. Based on computational modeling studies, these properties of the N400 component might correspond to a presumed transient over-activation within the semantic system. This could, for instance, be driven by a frequency-of-use dependent activation of orthographic neighbors and may account for responses upon both words and pseudo-words^69,70^. In view of the diverse findings referring to the N400, Kutas and Federmeier^28^ offered an abstract generalization in that the N400 reflects “activity in a multimodal long-term memory system that is induced by a given input stimulus during a delimited time window as meaning is dynamically constructed” (p. 640). This concept is compatible with a framework of language comprehension in which prediction occurs at multiple levels of representation with the goal to infer the intended message^51^. Such an incremental process includes context-driven prediction as well as the integration of linguistic information provided by single words into multi-word utterances, and has also been referred to as ‘semantic unification’^71^. A mismatch between contextual prediction and word recognition triggers the allocation of additional resources for semantic integration, which is indexed by increased N400 amplitudes^72^. The time-course of the N400, however, has been found to be rather independent from word recognition^73^, suggesting that semantic integration is initiated almost automatically upon hearing a word (or word-like utterance) even before its identification, cf.^74^. In this view, any word-like stimulus presented with linguistic context recruits semantic integration processing without necessarily having semantic content itself. Against this background, a possible interpretation of our current results in older adults could be that a diminished N400-effect upon phonemic (word and pseudo-word) priming in particular indicates stronger recruitment of additional resources to disambiguate words from pseudo-words. Increased use of linguistic context and thus predictive processing in particular might compensate difficulties during bottom-up analysis of auditory stimuli due to reduced hearing acuity in older adults^19,20^. This compensatory strategy has been shown to mitigate sensory decline, yet it also causes increased cognitive effort and results in higher processing costs^75,76^.

Several functional imaging studies have demonstrated altered information processing in brain structures involved in semantic cognition, i.e., linguistic and non-linguistic tasks that demand the use of semantic knowledge with increasing age; for a recent meta-analysis see^77^. It has been suggested that the ability to use neural resources during demanding cognitive tasks does not simply decrease with age, but shows altered activation within the semantic network, which might be due to less specific or selective activity—a phenomenon termed ‘dedifferentiation’^78^. This hypothesis suggests that particular cognitive capacities emerge from a distributed default network in childhood (differentiation) before a gradual loss of specialization (dedifferentiation) occurs later in life^79^. In particular, dedifferentiation affects fluid earlier than crystallized abilities, leading to increased coupling between intellectual abilities and cognitive processes^80,81^. Consistent with this idea, older adults exhibited increased activity in ‘domain-general’ brain regions associated with different cognitive domains implicated in task-related processing^77^. With respect to lexico-semantic access specifically, a similar pattern of age-dependent changes in brain activity as well as altered hemispheric specialization has been interpreted as indicating compensatory changes involving increased effort^82^. In contrast to functional imaging data, there is only very limited information about a possible link between dedifferentiation and ERP-changes with increasing age, and this has to our knowledge not yet been discussed with respect to altered N400 processing in older adults. Of course, due to the low spatial resolution of EEG, it is difficult to relate findings of altered brain activations observed in functional imaging to the pattern of N400 modulations in our current as well as in earlier studies. However, dedifferentiation in older adults has previously been related to reduced amplitude modulation of late posterior positive potentials such as the P3 component^83,84^. This pattern resembles the age-related N400 changes, since in both cases older adults did not show an overall decline of ERPs^58,59^, but rather absent or smaller reductions of amplitudes to one of the contrasting experimental conditions.

To conclude, we found an age-dependent N400 modulation by lexical context at word level in semantic and phonemic priming of words and pseudo-words which resembled the pattern obtained at sentence level in previous studies. Whereas the behavioral results pointed to even greater sensitivity to (or need for) context in older adults, the latter expressed markedly smaller N400 amplitude reductions accompanying phonemic and semantic priming than younger adults. This dissociation of behavioral and ERP results in older adults seems most reasonably to reflect age-related changes of resource allocation implying higher cognitive effort during lexical decisions, but it does not support a link between absent N400 effects and impaired use of lexical context. As dedifferentiation of cognitive abilities with age has been associated with changing patterns of intra- and interhemispheric brain activations in functional imaging studies, a correlation with altered N400 processing is a possible interpretation of the results that requires further investigation.

## Methods

### Participants

The experiment was evaluated and approved by the local ethics committee of the Charité – Universitätsmedizin Berlin (EA2/047/10). All participants gave their informed and written consent prior to the experiments and the research was conducted in accordance to current guidelines and the Declaration of Helsinki.

Thirty-six healthy individuals with normal or corrected-to-normal vision and unimpaired hearing participated in the study. Two EEG-recordings had to be discarded due to excessive eye or movement artifacts, thus the final data-analysis included a total of 34 participants of two age groups between 55 and 77 years of age (n = 15) and 23 and 46 years (n = 19), i.e., with an age cut-off set at 50 years. We assessed the overall cognitive state based on the Parkinson Neuropsychometric Dementia Assessment (PANDA)^85^. The PANDA was included because the older participants were (as a control group) part of a series of studies in patients with Parkinson’s disease. In addition, we evaluated semantic and phonemic verbal fluency (VF) by means of the German standard VF test^86^. The latter included both a non-alternating and an alternating task, where participants were instructed to produce as many words as possible belonging to the given categories within a 2-min time period: *vegetables* (semantic non-alternating), *animals* and *furniture* (semantic alternating), words beginning with the letter *S* (phonemic non-alternating), or *G* alternating with *R* (phonemic alternating).

Age groups were compared by using the χ^2^-test for dichotomous data (*sex, handedness*) and the Wilcoxon rank-sum or two-tailed t-test for non-dichotomous data (*age, education, PANDA-scores, VF scores*) according to distribution of the data indicated as by the Shapiro-Wilks test. VF output was compared by entering the individual word counts into a mixed repeated measures ANOVA with *task condition* (semantic / phonemic) and *task alternation* (alternating / non-alternating) as within-subjects factors and age *group* (old / young) as between-subjects factor.

Both groups did not differ with respect to sex (female/male: old 9/6 vs. young 9/10) or handedness (right/left: old 14/1 vs. young 17/2). The difference of age was, as expected, significantly different between both groups (*p* < 0.001): median age in the older group was 69.0 ± 7.2 years (range 55–77 years) in contrast to 29.0 ± 5.6 years (range 23–46 years) among the younger participants. Mean years of education were 10.6 (± 1.76) years in the elderly and 12.7 (± 0.48) years in the young group (*p* = 0.001). This difference cannot be avoided, since it reflects the increasing access to education throughout the twentieth century in Germany with an average number of school years of around 10 years (9.66 and 10.79) in the 1960s and 1970s and 12 years (11.76 and 12.45) in the 1980s and 1990s^87^, in line with the development in other industrialized countries. The cognitive evaluation of the participants did not reveal any significant differences of PANDA scores (old: 25.33 ± 3.5, young: 27.26 ± 3.4; *p* = 0.113). VF output did not differ between groups (factor *group*: *p* = 0.569) and there was no significant interaction between *group* and any other factors. Averaged across VF tasks and groups, participants produced 24.9 ± 5.6 words (old: 24.3 ± 6.5, young: 25.4 ± 4.8), with more words produced during phonemic (all: 25.9 ± 8.3; old: 25.6 ± 10; young: 26.1 ± 6.9) than semantic (all: 23.9 ± 8.3, old: 22.9 ± 4.3; young: 24.6 ± 4.2) VF tasks.

### Experimental procedures

During EEG-acquisition, participants were presented with auditory stimuli. The LDT required the differentiation of words from pseudo-words (each 45), presented as target-stimuli following word primes. Each of the 90 trials started with a fixation cross centrally presented on a 17-inch computer screen for 750 ms preceding the auditory presentation of the prime-word (mean duration of prime-stimuli was 745 ± 111 ms). Following an inter-stimulus-interval of 100 ms, the target-stimulus (with a mean duration of 746 ± 100 ms) was presented. The order of the trials was pseudo-randomized and counterbalanced across participants. The participants were instructed to press a response-button upon real words as targets only, whereas pseudo-words demanded no reaction. Prime-stimuli did not demand any response; the participants were not informed about a possible prime-target relatedness prior to the tasks. Target real words were either unrelated (15 trials), semantically related (15 trials), or phonemically related (15 trials) to the preceding primes. Phonemically related word pairs rhymed, meaning that their last phonemes were identical. Semantic relatedness was based on ratings of word-pairs on a 0–4 scale (0 = no relation; 4 = high relation) obtained from 50 healthy adults not participating in this study, confirming the definition of semantic relatedness in our set of stimuli; for further details of the methods see also^88,89^. Target pseudo-words were either phonemically related (15 trials) or unrelated (30 trials) to the preceding primes. The latter category contained 30 rather than 15 items since it is impossible to create semantically related pseudo-words. Like this, word/word and word/pseudo-word pairs were equiprobable. Altogether, five lists of targets (i.e., unrelated, semantically related, and phonemically related real words, phonemically related and unrelated pseudo-words) each encompassing 90 items were created to match the 90 prime words. By means of computerized pseudo-randomization the different prime-target constellations were counterbalanced across the participants.

All word-stimuli were mono- or disyllabic and did not differ between task-categories in the average number of letters, syllables, mean stimulus duration, or mean frequency of occurrence based on the database dlexDB^90^ (https://www.dlexdb.de). Pseudo-words were created as pronounceable and word-like by composing existing German phonemes, obtaining an equal number of letters and syllables as in the word stimuli.

The recorded stimuli were spoken by a trained female voice in neutral tone. During the experiment, they were presented at individually adjusted volume levels using semi-open earphone (BEYERDYNAMIC, DT-880). We used the PRESENTATION software (Version 15.0, Neurobehavioral Systems Inc.) for task presentation and result recording. EEGs were recorded from 14 scalp electrodes (F3, Fz, F4, C3, Cz, C4, P3, Pz, P4, O1, Oz, O2, T7, T8) positioned according to the 10–20 system with linked mastoid-electrodes as reference; impedances were < 5 kΩ. EEG-data was continuously sampled at a rate of 2 kHz using high and low-frequency band-pass filters at 0.05 and 500 Hz (amplifier: NEUROSCAN SYNAMPS2, software: ACQUIRE 4.5; Neuroscan, Charlotte, USA). Vertical and horizontal electro-oculograms were recorded for the off-line correction of eye-blink artefacts. Before the experiment, all participants completed test runs to become familiar with the task.

### Data analysis

#### ERP data

Analysis of EEG-data was performed using the software *Vision Analyzer* Version 2.0.1 (Brain Products GmbH, Germany). EEG-data was filtered from 0.1 to 20 Hz (12 dB/oct) and for the 50 Hz notch. DC detrending and ocular correction independent component analysis (ICA) were applied to the data before segmentation into epochs from −500 to 2000 ms time-locked to the onset of target-stimuli. A semi-automated algorithm was used for artifact removal based on blinks occurring within 1 s after target presentation, gradients of more than 30 µV or voltage exceeding ± 200 µV. The baseline was defined as the 100 ms segment before stimulus-presentation. ERPs were averaged per target stimulus class (words vs. pseudo-words), and relatedness (unrelated words and pseudo-words, semantically or phonemically related words, phonemically related pseudo-words). The N400 was analyzed based on the mean ERP voltage in a time-window between 350 and 700 ms post-stimulus based on visual inspection of grand averages across all participants and target stimuli, e.g.^47^. Based on the literature on the N400 effect and visual inspection of grand-averages, effects of prime-target relatedness were analyzed at midline frontal, central, and posterior electrodes (Fz, Cz, Pz). N400 latency was analyzed by using the fractional area method^91–93^. To this end, N400 latency was defined as the last time-point at which the calculated N400 area was < 50% of the total N400 area; individual boundaries from which the N400 area was integrated were chosen so signal fluctuations did not cross the 0-µV baseline.

#### Behavioral data

Lexical decision times were defined as reaction times (RTs), i.e., the time between onset of target-words and button-press upon correctly identified real words. With respect to the individual trials, we used the Grubb’s test to identify outlier data and to classify trials with unusually long RTs as errors. Only correct trials were further analyzed. This included RTs upon unrelated as well as semantically or phonemically related word-pairs. Priming effects were calculated by subtracting RTs upon related from RTs upon unrelated target words. Finally, we examined task accuracy as reflected by error rates (overall as well as by task condition). Furthermore, false responses upon pseudo-words (commission errors) and missed responses upon real words (omission errors) were analyzed separately.

#### Statistical analysis

Statistical analysis was conducted with mixed repeated measures analyses of variance (ANOVA) with *group* (old / young) as between-subjects factor in all analyses and within-subjects factors defined as the following: For RTs, within-subjects factors were *relatedness* (unrelated / related) and for priming effects obtained from subtraction of RTs *priming* (semantic / phonemic). The analysis of ERP mean voltage and fractional area latency was conducted separately for semantic and phonemic priming and thus included *group* as between-subjects factors (as above) for both conditions and for semantic priming *relatedness* (unrelated / related) as the only dependent variable, whereas phonemic priming (as it was studied in target real words and pseudo-words) additionally included the within-subject factor *stimulus category* (word / pseudo-word). Data that was not normally distributed as indicated by the Shapiro-Wilks test (error-rates) were therefore compared with non-parametric tests (i.e., Wilcoxon signed-rank and rank-sum tests). For both ERP and behavioral data only trials with correct responses were included in the further analysis.

We report F-values, degrees of freedom (df), *p*-values, and partial eta-squared (η_p_^2^) for an estimation of effect sizes^94,95^. The significance level for all statistical tests was *p* < 0.05. Significant interactions in the ANOVAs were decomposed by using paired *t*-tests or independent-samples *t*-tests using Bonferroni-adjusted significance thresholds as indicated. We used IBM SPSS STATISTICS version 25 for all statistical analyses.
